# Modulated anti-VEGF therapy under the influence of lipid metabolizing proteins in Age related macular degeneration: a pilot study

**DOI:** 10.1038/s41598-021-04269-6

**Published:** 2022-01-13

**Authors:** Kaushal Sharma, Priya Battu, Ramandeep Singh, Suresh Kumar Sharma, Akshay Anand

**Affiliations:** 1 Neuroscience Research Lab, Department of Neurology, Post Graduate Institute of Medical Education and Research, Chandigarh, India; 2 Advanced Pediatrics Centre, Department of Pediatrics, Post Graduate Institute of Medical Education and Research, Chandigarh, India; 3 Advanced Eye Centre, Department of Ophthalmology, Post Graduate Institute of Medical Education and Research, Chandigarh, India; 4 Department of Statistics, Panjab University, Chandigarh, India

**Keywords:** Epigenetics, Gene regulation, Genetic interaction, Medical genetics

## Abstract

Age-related macular degeneration (AMD) is a devastating retinal disease that results in irreversible vision loss in the aged population. The complex genetic nature and degree of genetic penetrance require a redefinition of the current therapeutic strategy for AMD. We aimed to investigate the role of modifiers for current anti-VEGF therapy especially for non-responder AMD patients. We recruited 78 wet AMD cases (out of 278 AMD patients) with their socio-demographic and treatment regimen. Serum protein levels were estimated by ELISA in AMD patients. Data pertaining to the number of anti-VEGF injections given (in 1 year) along with clinical images (FFA and OCT) of AMD patients were also included. Visual acuity data (logMAR) for 46 wet AMD cases out of a total of 78 patients were also retrieved to examine the response of anti-VEGF injections in wet AMD cases. Lipid metabolizing genes (LIPC and APOE) have been identified as chief biomarkers for anti-VEGF response in AMD patients. Both genotypes ‘CC’ and ‘GC’ of LIPC have found to be associated with a number of anti-VEGF injections in AMD patients which could influence the expression of B3GALTL,HTRA1, IER3, LIPC and SLC16A8 proteins in patients bearing both genotypes as compared to reference genotype. Elevated levels of APOE were also observed in group 2 wet AMD patients as compared to group 1 suggesting the significance of APOE levels in anti-VEGF response. The genotype of B3GALTL has also been shown to have a significant association with the number of anti-VEGF injections. Moreover, visual acuity of group 1 (≤ 4 anti-VEGF injections/year) AMD patients was found significantly improved after 3 doses of anti-VEGF injections and maintained longitudinally as compared to groups 2 and 3. Lipid metabolising genes may impact the outcome of anti-VEGF AMD treatment.

## Introduction

Degenerative changes of macular photoreceptors (rod and cones) can lead to irreversible vision loss in aged population. Age related macular degeneration has been associated with 52 independent genetic variants and various environmental factors like smoking, age, food habits, comorbidities^1,2^. Recently, our data has also indicated that association of sleeping pattern and activities of daily living with AMD which can stimulate the pathological changes by modulating protein expression^3^. Despite growing knowledge of AMD genetics, not much advancement in treatment of AMD has been noted in the field. Currently, anti-VEGF injection is prescribed for wet AMD patients in order to offer symptomatic relief to increasing visual acuity^4^. However, current therapies for both dry (vitamin supplementations) wet AMD (anti-VEGF injection) have been reported to retard the photoreceptor degeneration. Short term safety of intravitreal bevacizumab with an average of 2–3 injections per 3 months with a maximum of 4 injections was also investigated^5^. This has shown significant improvement in retinal thickness, analyzed by OCT along for visual acuity^6^. Withdrawal of bevacizumab therapy has been found to enhance the chance of recurrence of wet AMD by 10% every successive year^7^. Dose Optimization and frequency of Anti-VEGF injection can be influenced by genetic variants and the interactions between them. Genetic variant of CCT3 gene rs12138564 has been coupled to improved outcome of anti-VEGF treatment. On the contrary, the results from same study have also revealed a decreasing anti-VEGF response under the influence of rare genetic variants of *C10orf88* and *UNC93B1* genes in wet AMD patients^8^. Our previous genetic investigation on genetics AMD on Indian patients has defined the biological significance of systemic inflammation^9–11^, impaired angiogenic mechanism^12–14^, oxidative stress^15^ which showed TLR3 independent^16^ aggravation of AMD pathology along with the substantial contribution of environmental factors. Exploring the genetic penetrance of rare and common genetic variants and their pathological implication under the influence of confounders can determine the genetic complexity and susceptibility of AMD^17^ which can influence the disease phenotype and treatment outcome. This is suggestive of possible association of genetic variation and the influence of environmental factors (with or without interactions) which may modulate the outcome and number of anti-VEGF treatment in AMD patients which can contribute in AMD management. This study also describes the genetic susceptibility towards the response of Anti-VEGF treatment in Indian AMD patients.

## Methodology

### Recruitments of participants

The study population comprised of 277 patients with AMD recruited from Advanced Eye Centre, PGIMER, Chandigarh, India. Analysis of Anti-VEGF response was carried out on 78 cases of active wet AMD. Although the patients were recruited prospectively, the data of 11 patients was retrieved (from same recruited patients) retrospectively to examine the number of anti-VEGF injections given in a year. Moreover, the data of visual acuity was retrieved for 46 AMD cases out of a total of 78 wet AMD patients recruited in the study. The written informed consent was obtained from all the participants after explaining the nature of study. The experimental protocols were approved by Institute Ethical Committee (IEC) (No: PGI/IEC/2005-06; dated: 23.07.2013), PGIMER, Chandigarh, India. The study adhered to the study protocol and conducted as per the ethical guidelines laid down by Institute Ethical Committee, PGIMER, Chandigarh, India. The participants were also asked about the history of prescribed medication for any ailment along with AMD pathology. The socio-demographic (SD) details including smoking, alcohol consumption, and food habits (prior or current) etc. were also noted.

### Treatment regimen of Anti-VEGF therapy

**Figure 1:**
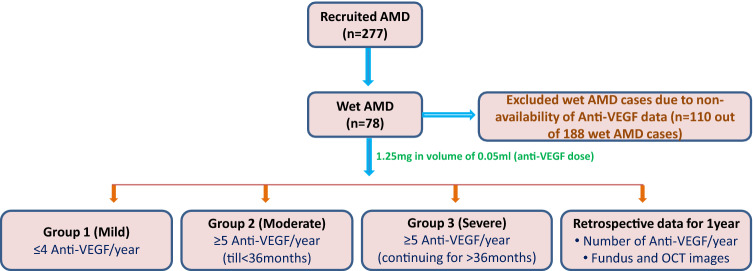
Schematic representation of groups categorised in study.

The details of a total number of anti-VEGF injections and an estimated duration of AMD pathology was obtained individually for each patient. Intravitreal Bevacizumab (1.25 mg/0.05 ml) was given to wet AMD patients. We categorised the wet AMD patients based on number of anti-VEGF injections given as described in Fig. 1. We administered three monthly doses of Bevacizumab followed by *pro re nata* (PRN) treatment. However, strict PRN could not be followed up in many patients owing to financial, and other logistic reasons in our part of the world.

### Clinical details

Clinical severity and categorization of AMD was done by a retina specialist by recording the fluorescein fundus angiography (FFA) and optical coherence tomography (OCT) images. AREDS criteria were adopted to classify the AMD pathology in the population. Snellen’s best corrected visual acuity (VA; US feet 20/20) data of 46 wet AMD patients out of the total of 78 AMD cases was collected at three time points including first (baseline), third and final visit of AMD patients along with the total visit (in months) made to the Department of Ophthalmology, PGIMER, Chandigarh. VA values were converted to logMAR scale and were considered for final data analysis. We did not take into the account the type of CNV (Classic or Occult) in our wet AMD patients. This is the limitation of our study.

### Serum extraction

Blood sample of patients was collected in Sodium citrate vacutainer and kept at room temperature for 1–2 h. Samples were centrifuged at 1800 rpm for 20–30 min at room temperature. Upper layer sample (serum) were collected and stored in − 80 °C for further experimental uses.

### Genomic DNA extraction

Genomic DNA from peripheral blood mononuclear cells (PBMCs) was extracted using commercially available kit (Qiagen, USA) to perform the SNP analysis. DNA was stored at − 20 °C till conducting the experiments.

### Total protein estimation

Bradford’s method was adopted to estimate the total protein levels in the patient’s serum. Briefly, diluted serum (600 times) was mixed with diluted Bradford’s reagent (1:4 ratio). Absorbance of the reaction was taken at 595 nm using ELISA reader (BioRad, USA).

### Retrospective analysis

In order to understand the response of anti-VEGF injections in different AMD phenotypes, we retrieved the clinical data of AMD patients (n = 11) including the number of anti-VEGF shots and clinical images (both FFA and OCT) in 1 year of duration.

### SNP analysis

Single nucleotide polymorphism (SNP) analysis was carried out for lipid metabolizing genes like LIPC (rs920915) and APOE (rs769449), pro-angiogenic genes including ADAMTS9 (rs6795735) and TIMP3 (rs5749482), regulatory genes *e.g.* B3GALTL (rs9542236), IER3 (rs3130783), HTRA1 (rs11200638) and SLC16A8 (rs8135665, monocarboxylic transporter protein). SNP analysis was carried out on StepOne real time PCR (Applied Biosysystems Inc., Foster city, CA) by using Taq Man assay (ThermoFisher, USA) as per the manufacturer’s instruction. Briefly, genomic DNA (20 ng) and 5ul of Taqman master mix was taken in the 10 μl of total volume of reaction setup. FAM and VIC tagged probes, to discriminate the allelic variation in genome at particular site, was added to the reaction. Reaction without genomic DNA was considered as negative control. Analysis of raw data to demonstrate the allelic condition (homozygous dominant/recessive and heterozygous) was performed using *Genotyper* and *StepOne V2.0* softwares (Applied Biosysystems Inc., Foster city, CA).

### ELISA

Serum levels of lipid metabolizing (APOE and LIPC), pro-angiogenic (TIMP-3 and ADAMTS9), regulatory (HTRA1, IER3 and B3GALTL) and monocarboxylic acid transporter (SLC16A8) proteins were estimated by commercially available ELISA kits (Qayee Biological Technology Co. Ltd., Shanghai, China). Serum samples were diluted before performing the experiments. The protocol was followed as per the manufacturer’s instructions. Briefly, diluted serum samples were incubated with primary and secondary antibodies in dark at 37 °C for one hour. Washing was carried out 5 times, using 1X diluted washing buffer before adding the substrates to the reaction. Reaction was terminated by adding stop solution followed by estimation of absorbance at 450 nm in ELISA reader (BioRad, USA). The values were further neutralized with total protein levels for respective patients.

### Statistical analysis

Comparative analysis of protein expression between various groups was estimated using One-way ANOVA, independent *T*-and Mann–Whitney tests. Pearson’s chi square analysis was applied to reveal the association between number of anti-VEGF treatment and genotype frequency of various SNPs along with SD parameters. Logistic regression analysis was carried out to study the association of number of anti-VEGF shots and protein expression. Moreover, changes in protein expression with respect to single nucleotide polymorphism (for respective gene) were also analysed using contrast analysis with or without controlling anti-VEGF numbers. Wilcoxon sign-ranked test was employed to compare the changes in visual acuity of AMD patients throughout treatment regimen. Multivariate model analysis was performed to understand the effect of genotype interactions on anti-VEGF response (number of anti-VEGF injections per year). Survival curve was also generated for current data set in order to show direct relationship between number of anti-VEGF and progression of AMD pathology. Z-proportions test was applied to compare minor allele frequency (MAF) derived from GAW studies (INDEX-DB and IndiGenomes) conducted on Asian population with current study.

## Results

### Association of anti-VEGF injections with socio-demographic details

Results of *chi-square* suggest that alcohol addiction could be a modulator for anti-VEGF response in Indian AMD patients. Similarly, AMD patients with history of cataract surgery (single or both eyes cataract surgery) can also significantly alter the anti-VEGF response. Both results indicate the complex nature of AMD pathology where activities of daily living and associated ailment could act as a modifier for anti-VEGF response in AMD (Table 1).

**Table 1:** Association of anti-VEGF response (based on number of anti-VEGF injections given during the course of disease) with daily living habits (Socio-demographic details) of AMD patients including alcohol consumption and cataract surgery in AMD patients.

	Status	Avastin response			Total	P-value
		Mild	Moderate	Non-responsive		
Alcohol habit	Never	37	9	5	51	**0.024**
	Past	5	0	2	7	
	Current	8	8	1	17	
Total		50	17	8	75	
Cataract surgery	No surgery	29	8	3	40	**0.018**
	One eye surgery	22	8	3	33	
	Both eyes surgery	0	1	2	3	
Total		51	17	8	78	

Mild- < 4 Avastin/year; Moderate- ≥ 5 Avastin/year; Non-responsive- ≥ 5 Avastin/year and continuous for > 36 months.

### Genotype influences anti-VEGF response in AMD pathology

Chi-square analysis has revealed a significant association of B3GALTL and LIPC variants with anti-VEGF response in Indian AMD patients. Results demonstrate that the frequency of homozygous ‘CC’ and heterozygous ‘CT’ of B3GALTL are more frequent in AMD patients, being moderate and non-responsive towards anti-VEGF response with context to number of injections given to the patients. Similarly, both homozygous ‘CC’ and heterozygous ‘GC’ genotypes of LIPC are also associated with number of injections given to AMD patients (Table 2). A complex nature of AMD pathology due to its heterogeneity and genetic interaction along with equal contribution of environmental factors has been widely investigated which has also been supported by our data. However, we did not find significant association of remaining genotypes with the number of anti-VEGF injections given to the wet AMD patients (Table S1).

**Table 2:** Association of genotypes of (Pearson’s *Chi*-square) B3GALTL (rs9542236) and LIPC (rs920915) with number of anti-VEGF injections given to AMD patients to demonstrate the genetic susceptibility of both genes towards response of anti-VEGF treatment in AMD pathology.

	Genotypes	Anti-VEGF response			Total	P-value
		Mild	Moderate	Non-responsive		
B3GALTL Genotype (rs9542236)	Homozygous TT	32	6	2	40	0.033
	Homozygous CC	1	0	1	2	
	Heterozygous CT	13	9	2	24	
Total		46	15	5	66	
LIPC genotype (rs920915)	Homozygous GG	18	8	1	27	0.013
	Homozygous CC	0	2	2	4	
	Heterozygous GC	25	5	4	34	
Total		43	15	7	65	

Mild- < 4 Avastin/year; Moderate- ≥ 5 Avastin/year; Non-responsive- ≥ 5 Avastin/year and continuous for > 36 months.

### Comparison of minor allele frequency derived from Asian GWAS studies

We have compared the minor allele frequencies (MAF) of studied genes with GWA studies conducted especially on Asian (INDEX-DB) and Indian (IndiGenomes) population by considering the fact of small sample size for final analysis in current study. Results of Z-test proportions did not show significant alteration of MAF between IndiGenomes and current study except *HTRA1* (Table 3). Our study has indicated that response of anti-VEGF injections was found to be varied based on *LIPC* genotype and the level of APOE. We did not find frequencies of minor alleles of the studies genes in INDEX-DB except *APOE* gene which was found to be similar as frequency shown in IndiGenomes. However, references genomes from both studies haven’t assessed the effect of different genotypes on anti-VEGF response or any kind of treatment strategies.

**Table 3:** Comparison of minor allele frequency derived from IndiGenome and INDEX-DB GWAS with current study.

Genotype	Allele	MAF frequency current study	MAF from IndiGenome	MAF from INDEX-DB	P-value
B3GALTL (rs9542236)	C	28 (0.21)	0.18	NA	0.41
LIPC (rs920915)	G	88 (0.67)	0.73	NA	0.38
ADAMTS9 (rs6795735)	T	95 (0.73)	0.77	NA	0.49
APOE (rs769449)	A	9 (0.07)	0.08	0.083 (GnomAD)	0.71*
HTRA1 (rs11200638)	A	86 (0.67)	0.34	NA	< 0.001
TIMP3 (rs5749482)	C	12 (0.08)	0.15	NA	0.15
IER-3 (rs3130783)	A	111 (0.91)	0.91	NA	0.99
SLC16A8 (rs8135665)	T	34 (0.27)	0.19	NA	0.13

MAF: Minor allele frequency; *p-value based on comparison between IndiGenome and current study.

### LIPC genotype influences protein expression

**Figure 2:**
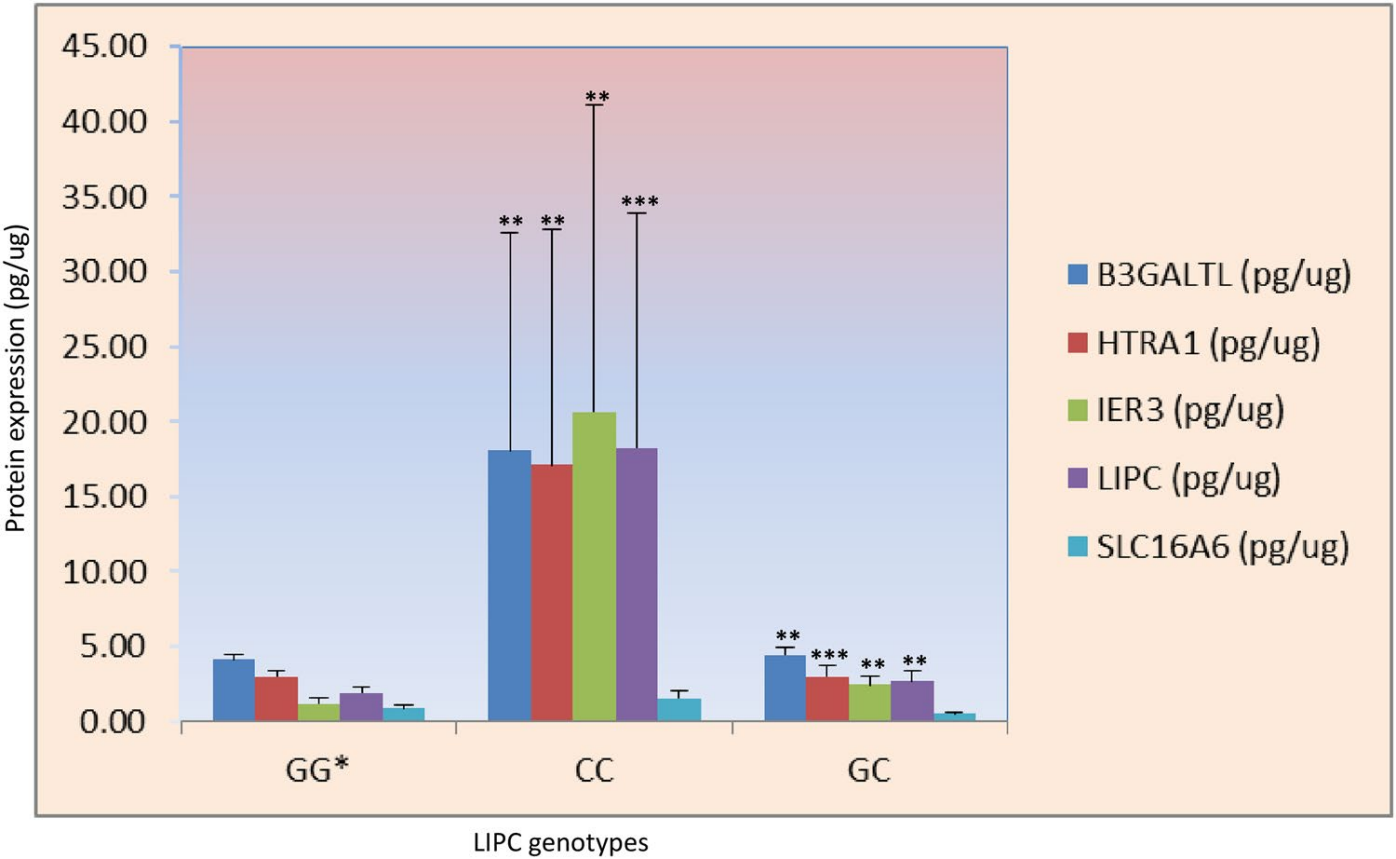
Impact of LIPC genotype on protein expression. Significant elevated expressions of B3GALTL, HTRA1, IER3 and LIPC were seen in ‘CC’ genotype of LIPC genetic variant (rs920915) as compared to both reference ‘GG*’ and heterozygous ‘GC’ alleles. GG* Reference allele. Bar is representing SEM; P < 0.05.

Associated genotypes of LIPC with anti-VEGF numbers have also been found to influence the majority of protein expression analysed in the study. We have demonstrated that homozygous ‘CC’ genotype of LIPC variant show enhanced expression of regulatory (HTRA1, B3GALTL and IER3), monocarboxylic transporter protein SLC16A8, and levels of LIPC itself. Moreover, significant alteration of protein expression, including HTRA1, IER-3 and LIPC, has also been examined in heterozygous ‘GC’ genotype of LIPC variants (Fig. 2). However, we did not find significant alteration of proteins among B3GALTL genotypes which has also showed the association with number of anti-VEGF injection in AMD patients (Table 2). Similarly, the expression of studied proteins were not found to be significantly altered with reference to other genotypes except the SLC16A8 expression between ‘AA’ and ‘GA’ genotypes of HTRA1 (Table S2).

Additionally, contrast estimate indicated significant changes in LIPC levels by 17.578 pg/ug with alteration of genotype i.e. from ‘GG (reference genotype)’ to ‘CC’ genotype (p = < 0.0001) which is consistent with our previous results^15^ (Table 4). Interestingly, we did not find any significant alteration for any other protein levels against the changes in genotypes (of studied variants) while considering anti-VEGF number as covariate. Results show an indirect role of lipid metabolism by regulating the action of associated proteins (LIPC) in controlling the anti-VEG response. Results also signify the biological significance of particular genotype (of variants), genetic and allelic interactions under the influence of confounders which may influence the various protein expressions thereby modulating the AMD treatment outcome after anti-VEGF.

**Table 4:** Contrast estimate to see the impact of genotype and response of anti-VEGF in AMD. Contrast estimate indicates the significant of per unit change in genotype (nucleotide/polymorphism) from ‘GG’ (reference genotype) to ‘CC’ in LIPC genetic variant (rs920915) by alteration the LIPC levels (17.58 pg/unit changes).

Genotype	Genotypes	Significant genotypes^+^			After controlling Anti-VEGF numbers				
		Contrast estimate	SE	p-value	B	SE	t-value	p- value	95% CI
ADAMTS9 (pg/ug)	CC vs. TT*	− .358	4.585	.938	.030	.139	.213	0.83	− 0.249–0.309
	CT vs. TT*	2.321	2.471	.352					
APOE(pg/ug)	AA vs. GG*	.001	.002	.732	0.00002	0.00006	.364	0.72	− 0.0001–0.00015
B3GALTL (pg/ug)	CC vs. TT*	− 4.770	7.311	.517	.062	.124	.499	0.62	− 0.186–0.309
	CT vs. TT*	− 2.260	1.910	.242					
HTRA1 (pg/ug)	AA vs. GG*	.512	2.168	.814	− .003	.098	− .030	0.98	− 0.199− 0.193
	AG vs.GG*	− 0.689	2.253	.786					
LIPC (pg/ug)	CC vs. GG*	17.578	3.972	< 0.0001	− .131	0.100	− 1.314	0.19	− 0.332–0.070
	CG vs. GG*	0.827	1.801	.648					
TIMP3 (pg/ug)	CC vs.GG*	0.011	0.011	.327	− 0.0002	0.00048	− .320	0.75	− 0.001–0.001
	GC vs. GG*								
IER-3 (pg/ug)	GG vs. AA*	2.045	3.834	.596	0.032	.153	.209	0.83	− 0.277–0.341
	AG vs. AA*								
SLC16A8(pg/ug)	TT vs. CC*	− 1.020	.638	.116	0.004	0.015	.285	0.77	− .025–0.034
	TC vs. CC*	− .410	.247	.103					

Alteration in expression levels with reference by changing in nucleotides (‘GG’ to ‘CC’) didn’t show any alterations indicating the indirect implication of anti-VEGF injections in AMD pathology (by considering the anti-VEGF numbers as covariate).

### APOE mediated anti-VEGF response in AMD

**Figure 3:**
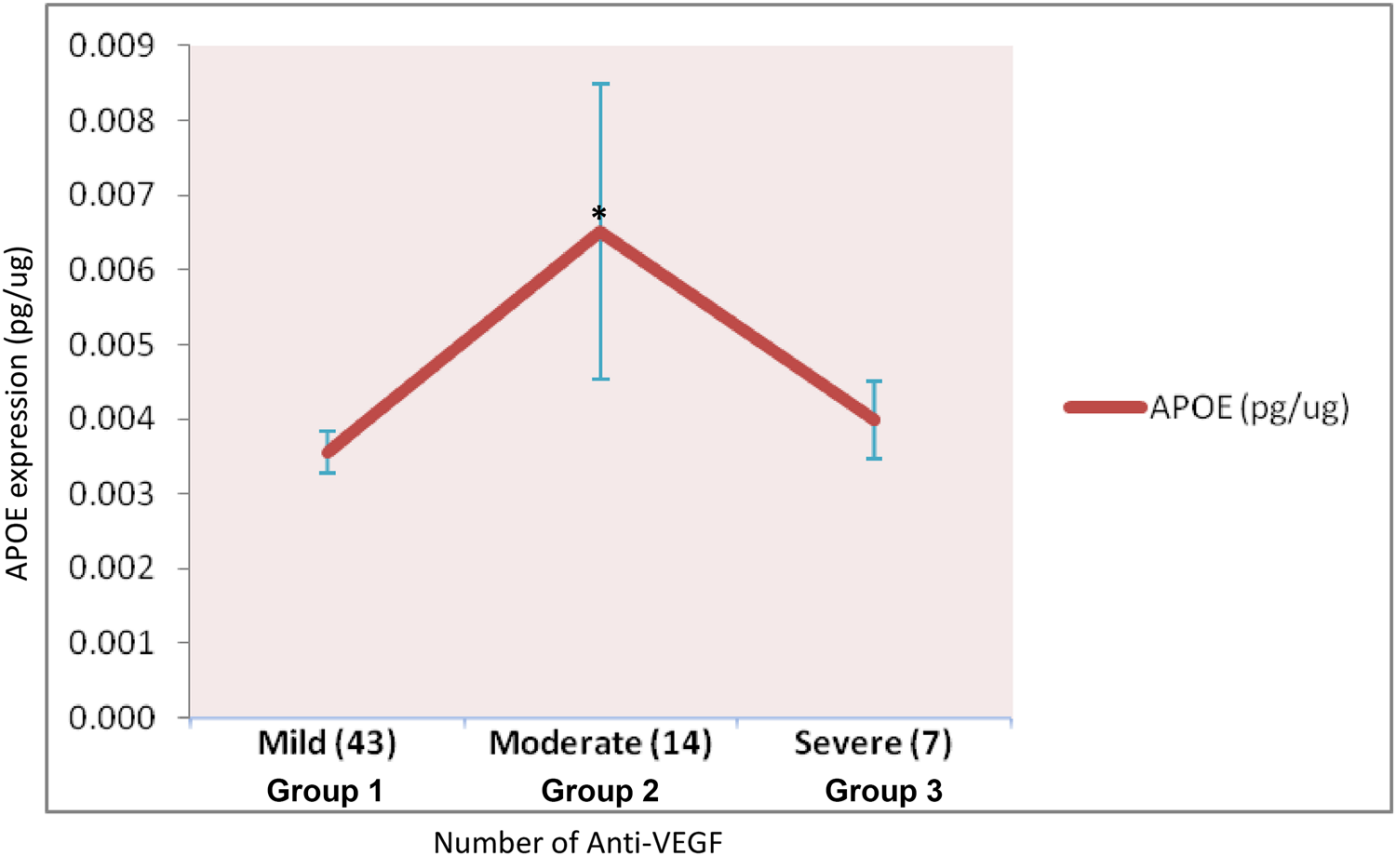
APOE expression in mild, moderate and severe groups of anti-VEGF response is based on the number of injections in wet AMD patients. Significantly higher levels of APOE were seen in moderate group as compared to mild group. Bar is representing SEM; P < 0.05.

Enhanced APOE levels with successive anti-VEGF injections (≥ 5 of per year) in AMD patients have suggested the APOE dependent anti-VEGF response in Indian AMD (Fig. 3). Significantly elevated expression of APOE has been observed in moderate group (group 2; ≥ 5 anti-VEGF injections/year and continuing for < 36 months) as compared to mild group (group 1; ≤ 4 anti-VEGF injections/year). Similarly, APOE levels were also found to be higher in severe group (group 1; ≥ 5 anti-VEGF/year and continuing for > 36 months) as compared to mild group of AMD, though it was not statistically significant. Results suggested that lipid metabolizing genes (especially APOE and LIPC) may modulate the action of anti-VEGF in AMD pathology.

**Figure 4:**
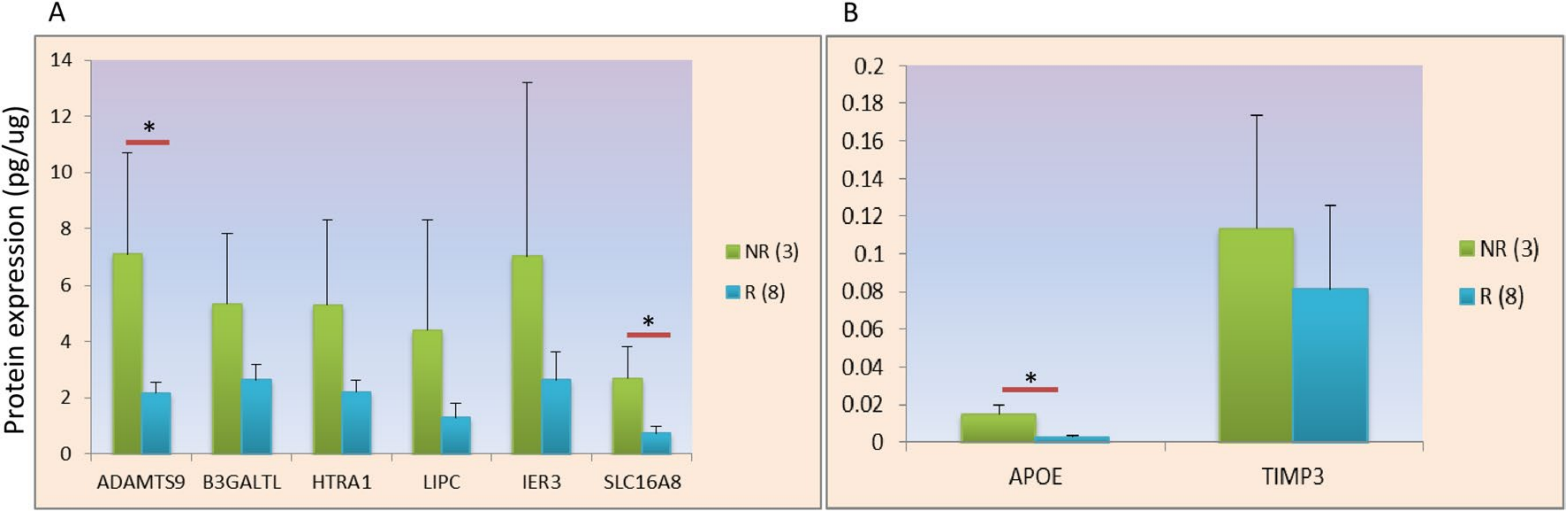
Differential expression of proteins in retrospectively group (Group 4). (**A**) Significant higher expression of ADAMTS9 and SLC16A8 in anti-VEGF non-responder (≥ 5 anti-VEGF injections/year), as compared to responders (≤ 4 anti-VEGF injections/year) in wet AMD patients. (**B**) APOE expression significantly higher in non-responder (≥ 5 anti-VEGF injections/year) for anti-VEGF AMD patients in comparison to responders (≤ 4 anti-VEGF injections per year). NR: non-responsive wet AMD for anti-VEGF treatment; R: responsive wet AMD for anti-VEGF treatment. Bar is representing SEM; P < 0.05.

To further validate the results suggesting the role of lipid metabolizing genes in anti-VEGF response, we assessed the scale of anti-VEGF injections given to AMD patients (for 11 AMD patients, Fig. 4). Pearson’s correlation analysis has revealed the positive correlation between anti-VEGF treatment and expression of ADAMTS9 (PCC = 0.629; P = 0.020), APOE (PCC = 0.872; P = < 0.0001) and SLC16A8 (PCC = 0.656; P = 0.014). Response of anti-VEGF treatment on AMD pathology in modulating the protein expression was further analysed and modelled by regression analysis to support the Pearson’s correlation results. Adjusted *Cox* and *Snell's* *R*^2^ values as 0.734 and 0.761, respectively were observed for logistic model. Regression analysis has demonstrated that APOE is significantly associated with anti-VEGF injections in a period of time (in one year) in Indian AMD pathology (Fig. 4 & Table 5). Results suggest that APOE and LIPC may act as chief modulator for anti-VEGF treatment in AMD patients.

**Table 5:** Logistic regression analysis to show the association of number of anti-VEGF injection on APOE expression in AMD pathology in retrospectively analyzed AMD patients.

Coefficients^a^							
Model	Unstandardized coefficients		Standardized coefficients	t	P-value	95.0% confidence interval for B	
	B	Std. error	Beta			Lower bound	Upper bound
Constant	.514	.423		1.215	0.255	− .443	1.470
APOE	251.530	47.041	.872	5.347	< 0.0001	145.116	357.945

^a^Dependent variable: anti-VEGF number.

When we compared the visual acuity data among studied groups, significant improvement of visual acuity from baseline was observed in group 1 AMD cases after three doses of anti-VEGF treatment as compared to group 2 and group 3. However, visual acuity was also improved in case of group 2 and 3 AMD cases after 3 doses of anti-VEGF treatment but it was non-significant. Number of anti—VEGF injections were further correlated with visual acuity (VA) of group-wise AMD patients along with their total follow up. Results have also shown that while comparing final visual acuity of group 2 and 3, AMD cases within group 1 worsened. Longitudinal follow-up of patients revealed more consistent results of visual acuity examined in group 1 AMD patients as compared to group 2 and group 3 (Table 6). This may require more anti-VEGF injections to stabilize the visual acuity as in case of group 2 and 3 in our results.

**Table 6:** Response of anti-VEGF treatment on visual acuity (logMAR) among different anti-VEGF groups of AMD patients (*i.e.* group 1, 2 and 3) and total follow-up (in months) during the course of disease.

Group	Mean ± SD logMAR						P-Value						Average follow up (months)
	Baseline VA		VA after 3 injections		Final VA		Left 1^st^ Vs 3^rd^	Right 1^st^ Vs 3^rd^	Left 1^st^ Vs final	Right 1^st^ Vs final	Left third vs final	Right third vs final	
	Left eye	Right eye	Left eye	Right eye	Left eye	Right eye							
Group 1 (n = 35)	0.95 ± 0.60	0.97 ± 0.50	0.75 ± 0.58	0.82 ± 0.61	1.07 ± 0.68	1.0 ± 0.63	0.003	0.007	0.334	0.807	0.025	0.225	65
Group 2 (n = 7)	0.92 ± 0.53	0.56 ± 0.24	0.59 ± 0.30	0.75 ± 0.26	1.49 ± .52	1.49 ± 0.64	0.109	0.665	0.357	0.180	0.144	0.180	75
Group 3 (n = 4)	0.33 ± 0.23	0.63 ± 0.17	0.28 ± 0.31	0.39 ± 0.26	1.25 ± 0.46	1.82 ± 0.13	0.317	0.109	0.109	0.066	0.109	0.068	103

### Influence of genetic interaction on anti-VEGF response

Our results have shown the role of lipid metabolizing genes in modulating anti-VEGF response in AMD pathology. Hence, we further attempted to assess the impact of genetic interaction on anti-VEGF response in AMD. The analysis of data revealed a significant genotype interaction among ADAMTS9-TIMP3 genes in AMD pathology. However, we did not find direct influence of genotype interaction on response of anti-VEGF treatment (in terms of number of injections given) and association with disease progression (Table 7). Results also suggest that studied SNP variants and their genetic interactions, especially among pro-angiogenic genotypes (ADAMTS9-TIPM3), may exacerbate the AMD pathology suggesting an indirect implication of the same on anti-VEGF response.

**Table 7:** Multivariate analysis to demonstrate genotype interaction of studied SNPs (based on their cellular functions) and influence of anti-VEGF treatment on AMD pathology. Results showed significant genotype interaction of pro-angiogenic genes including ADAMTS9 (rs6795735) and TIMP3 (rs5749482), but didn’t show direct influence of genotype interactions on number of anti-VEGF injections in Indian AMD patients.

Multivariate tests							
Genotype interactions	Effect	Test	Value	F	Hypothesis df	Error df	P-value
B3GALTL (rs9542236) * LIPC (rs920915)	Intercept	Pillai's Trace	.342	9.875	2	38	< 0.0001
	Anti-VEGF number	Pillai's Trace	.056	1.137	2	38	.331
	B3GALTL genotype	Pillai's Trace	.011	.103	4	78	.981
	LIPC genotype	Pillai's Trace	.475	6.078	4	78	< 0.0001
	B3GALTL * LIPC genotype	Pillai's Trace	.051	1.014	2	38	.372
APOE (rs769449) * HTRA1 (rs11200638)	Intercept	Wilks' Lambda	.260	58.273	2	41	< 0.0001
	Anti-VEGF number	Wilks' Lambda	.943	1.246	2	41	.298
	APOE genotype	Wilks' Lambda	.810	4.795	2	41	.013
	HTRA1 genotype	Wilks' Lambda	.781	2.695	4	82	.036
	APOE * HTRA1	Wilks' Lambda	.835	1.938	4	82	.112
Pro-angiogenic genotype interaction ADAMTS9 (rs6795735) * TIMP3 (rs5749482)	Intercept	Pillai's Trace	.698	46.138	2	40	< 0.0001
	Anti-VEGF number	Pillai's Trace	.006	.127	2	40	.881
	ADAMTS9 Genotype	Pillai's Trace	.408	5.260	4	82	.001
	TIMP3 genotype	Pillai's Trace	.370	11.751	2	40	< 0.0001
	ADAMTS9 * TIMP3 genotype	Pillai's Trace	.480	6.466	4	82	< 0.0001
Regulatory genotype interaction HTRA1 (rs11200638) * IER3 (rs3130783)	Intercept	Pillai's Trace	.189	4.090	2	35	.025
	Anti-VEGF number	Pillai's Trace	.035	.640	2	35	.533
	HTRA1 genotype	Pillai's Trace	.071	.666	4	72	.618
	IER3 genotype	Pillai's Trace	.002	.028	2	35	.972
	HTRA1 * IER3 genotype	Pillai's Trace	.033	.596	2	35	.557
Cellular function SLC16A8 (rs8135665) * B3GALTL (rs9542236)	Intercept	Pillai's Trace	.100	2.271	2	41	.116
	Anti-VEGF number	Pillai's Trace	.008	.175	2	41	.840
	SLC16A8 genotype	Pillai's Trace	.091	.998	4	84	.413
	B3GALTL	Pillai's Trace	.086	.941	4	84	.445
	SLC16A8 * B3GALTL	Pillai's Trace	.007	.146	2	41	.864
Lipid metabolizing APOE (rs769449) * LIPC (rs920915)	Intercept	Pillai's Trace	.324	8.871	2	37	.001
	Anti-VEGF number	Pillai's Trace	.013	.249	2	37	.781
	APOE genotype	Pillai's Trace	.006	.112	2	37	.895
	LIPC genotype	Pillai's Trace	.057	.553	4	76	.697
	APOE * LIPC	Pillai's Trace	.078	1.575	2	37	.221

**Figure 5:**
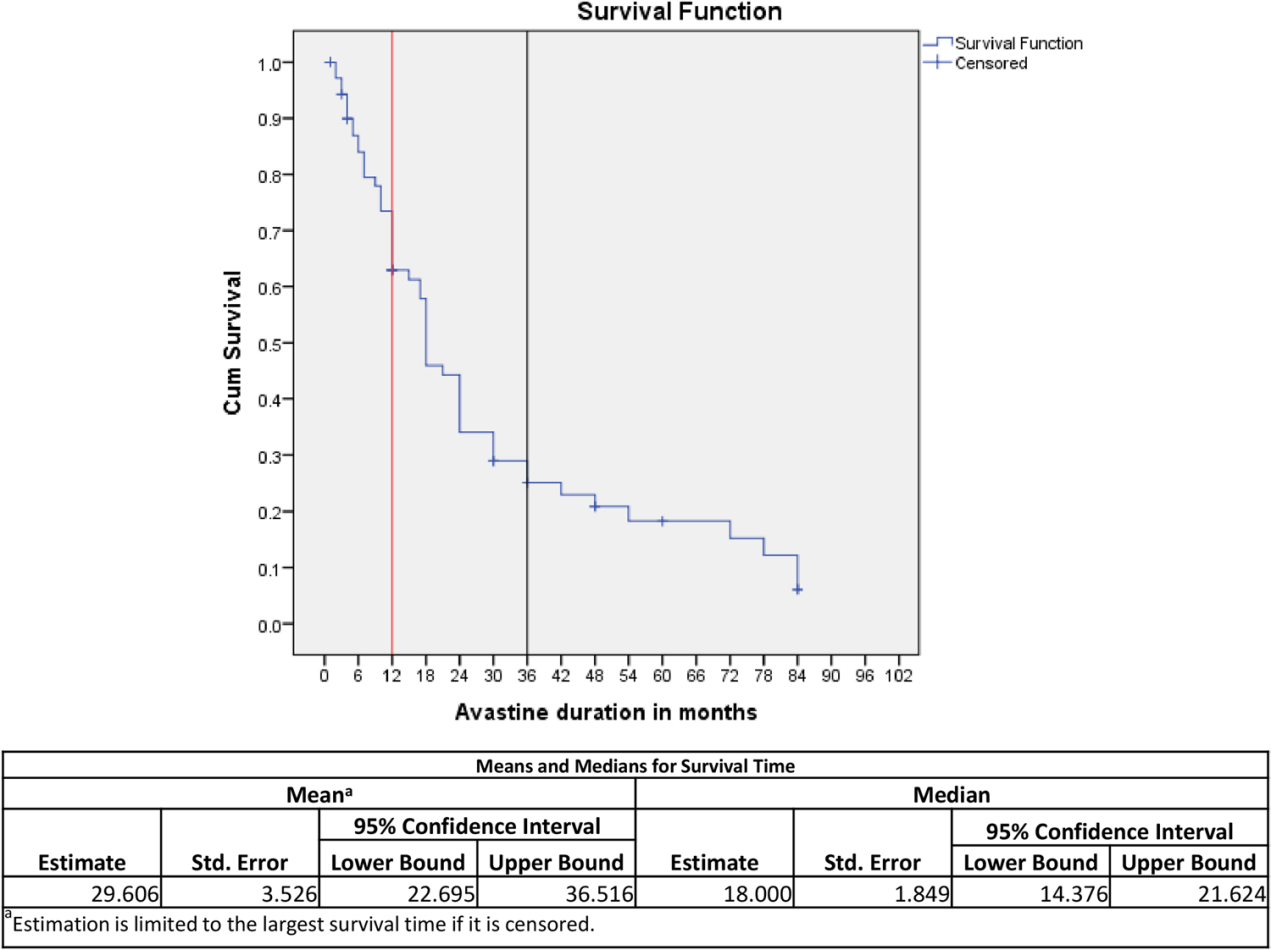
Survival curve to demonstrate the symptomatic recovery in wet AMD patients after treating with anti-VEGF injections during the course of disease.

We wanted to examine the progress of disease in patients as with the duration of disease (in months), such as the effect of anti-VEGF treatment, until the occurrence of the AMD pathology. For this purpose, survival analysis was performed and Kaplan–Meier survival curve revealed that at 12 months anti-VEGF treatment can provide 64% symptomatic recovery from AMD, while at 36 months, it was only 25% (Fig. 5). Subsequently, symptomatic relief from AMD by anti-VEGF treatment waned in patients receiving the successive anti-VEGF treatment with gradual increase in number of injections (anti-VEGF). This may be due to uncontrolled activity of lipid metabolizing proteins under the influence of confounders along with the genetic complexity of an individual^15^. Moreover, we have also determined the median survival time by locating the (time ‘in months’), at which the cumulative survival proportion is 0.5. In our study, median survival rate due to the effect of anti-VEGF treatment is 18 months with standard error of 1.849 and confidence intervals (14. 38–21.63) (Fig. 5).

## Discussion

The need for personalized medicine cannot be emphasised unless the genetics and nature of interactions with genetic variants and environmental factors well understood which acts as a roadblock towards translational approach in AMD genetics^18^. This study has attempted to understand the unique outcome of anti-VEGF treatment under the influence of confounders and genetic variants. We have shown the outcome of anti-VEGF (in context to number of injections given during the disease course) associated with both environmental (alcohol consumption and cataract history) and genetic factors (genetic variants of B3GALTL and LIPC). Poor response of Aflibercept has also been observed with higher BMI and geographic atrophy AMD patients^19^. Aqueous humor levels of angiogenic and pro-angiogenic proteins including VEGF-A, VEGF-C, interleukin 8, endothelin 1, HGF (Hepatocyte growth factor), HB-EGF (Heparin-binding epidermal growth factor-like growth factor), follistatin, and angiopoietin 2 were also found to be elevated after intravitreal injection of bevacizumab^20^. ATG haplotype of rs699947 (− 2578 C/A), rs2010963 (+ 405 C/G) and rs3025039 (+ 936 C/T) SNPs has been earlier shown to be associated with ‘poor’ responder of intravitreal bevacizumab in Tunisian AMD Patients^21^. Our results suggest that VEGF could be a potential identifier for anti-VEGF response by considering the lipid metabolizing genes as a modifier (especially APOE and LIPC) which is consistent with our previous report in the field^12^. Recently, TT genotype of CFH genetic variant (Y402H) was shown to increase the function and response of intravitreal ranibizumab in AMD patients^22^. Interestingly, a significant alteration in LIPC (lipid metabolizing), TIMP-3 (angiogenic) and SLC16A8 (monocarboxylic transporter) was observed in CFH negative AMD cases^23^. Our results have also revealed the association of genetic variants of B3GALTL and LIPC with the number of anti-VEGF injections in Indian AMD patients. Moreover, we also found a significant differential expression of B3GALTL, HTRA1, IER3 and LIPC proteins among subgroups of LIPC genotype. Genetic interaction of various genotypes can also influence the outcome of anti-VEGF treatment in AMD pathology. We have demonstrated a significant interaction between pro-angiogenic ADAMTS9-TIMP3 genotypes. However, we did not find significant association between number of anti-VEGF injections and such genetic interaction studied in our population. This indicate a complex nature of AMD pathology and associated response of anti-VEGF treatment which can be dependent on the nature of genetic interaction along with contribution of confounders^24^. Moreover, our results have also showed that both APOE and LIPC may act as biomarkers to differentiate degree of anti-VEGF response in wet AMD cases with respect to number of anti-VEGF injection given to the patients. The treatment strategy for lipid metabolism (by targeting APOE and/or LIPC) along with anti-VEGF may be a crucial step for effective management of AMD. Results of visual acuity and changes VA after anti-VEGF treatment have suggested the group 1 as a responder in comparison to group 2 and 3 where anti-VEGF treatment did not lead to significant changes in VA (especially after 3 doses of anti-VEGF injections). Out results of visual acuity and number of anti-VEGF injections have further supported the hypothesis of current study which indicates subsequent changes in number of anti-VEGF injections (or response) and visual acuity outcome based on genetic susceptibility of AMD patient.

Conclusively, results indicate the prominent biological significance of lipid metabolizing molecules (including APOE and LIPC) which may influence the anti-VEGF outcome in AMD patients. Impact of genetic variants and their interaction cannot be ignored in modulating the anti-VEGF response which must be considered for redefining the management of AMD pathology. However, conclusion of this study was drawn on limited number of samples along with number of anti-VEGF injections. Visual acuity of anti-VEGF treated groups has also suggested that group 1 AMD patients (≤ 4 anti-VEGF injections/year) respond to anti-VEGF treatment and showed more persistent visual acuity as compared to group 2 (≥ 5 anti-VEGF injections/year till < 36 months) and 3 (≥ 5 anti-VEGF injections/year for > 36 months). Final visual acuity of group 2 and 3 have further deteriorated than group 1 AMD cases indicating the longitudinal implication of genetic susceptibility (especially through LIPC and APOE) and response towards anti-VGEF treatment (also the number of anti-VEGF injections). This study could serve as substrate to design larger study on geographically diverse range of population based on their genetic susceptibility, genetic interactions, penetrance and influence of environmental factors.

## Supplementary Information

Supplementary Information.

## Data Availability

Whole data can be provided by first and corresponding authors of the manuscript without any restriction whenever required.
