# Supplementary material for: Modulated anti-VEGF therapy under the influence of lipid metabolizing proteins in Age related macular degeneration: a pilot study
Source: Sci Rep. 2022 Jan 13;12:714. doi: 10.1038/s41598-021-04269-6 (PMC8758686; doi:10.1038/s41598-021-04269-6)
Supplement: Supplementary file 1 — Supplementary Information. [file 41598_2021_4269_MOESM1_ESM.docx]

| Crosstab | | | | | |
| --- | --- | --- | --- | --- | --- |
|  |  | **Anti-VEGF response** | | | **P-value** |
|  | **Genotypes** | **Group 1** | **Group 2** | **Group 3** |  |
| ADAMTS9  (rs 6795735) | Homozygous TT | 23 | 8 | 4 | 0.869 |
|  | Homozygous CC | 3 | 2 | 0 |  |
|  | Heterozygous CT | 17 | 6 | 2 |  |
| Total |  |  |  |  |  |
| APOE  (rs 769449) | Homozygous GG | 38 | 14 | 6 | 0.956 |
|  | Heterozygous AG | 7 | 2 | 1 |  |
| Total |  | 45 | 16 | 7 |  |
| HTRA1  (rs11200638) | Homozygous GG | 10 | 2 | 1 | 0.711 |
|  | Homozygous AA | 22 | 9 | 4 |  |
|  | Heterozygous GA | 13 | 2 | 1 |  |
| Total |  | 45 | 13 | 6 |  |
| TIMP-3  (rs 5749482) | Homozygous GG | 35 | 15 | 6 | 0.344 |
|  | Heterozygous GC | 10 | 1 | 1 |  |
| Total |  | 45 | 16 | 7 |  |
| IER-3  (rs 3130783) | Homozygous AA | 34 | 12 | 5 | 0.062 |
|  | Homozygous GG | 0 | 1 | 0 |  |
|  | Heterozygous AG | 6 | 0 | 3 |  |
| Total |  | 40 | 13 | 8 |  |
| SLC16A8  (rs 8135665) | Homozygous CC | 18 | 9 | 4 | 0.231 |
|  | Homozygous TT | 3 | 0 | 0 |  |
|  | Heterozygous CT | 23 | 4 | 1 |  |
| Total |  | 44 | 13 | 5 |  |

Mild/group 1- <4 Avastin/year; Moderate/Group 2-≥5 Avastin/year; Non-responsive/Group 3- ≥5 Avastin/year and continuous for >36months

**Table S1:** Association of various genotypes with anti-VEGF treatment in North Indian AMD patients.

|  | ADAMTS 9 (rs rs6795735) Genotype | | | |  |  |
| --- | --- | --- | --- | --- | --- | --- |
|  | TT | CC | | CT | F-value | P-value |
| IER3 (pg/ug) | 3.84 ± 1.74 | 4.66 ± 1.97 | | 2.91 ± 0.58 | 0.153 | 0.858 |
| ADAMTS9 (pg/ug) | 4.99 ± 0.77 | 1.92 ± 1.07 | | 5.22 ± 2.39 | 0.348 | 0.708 |
| APOE (pg/ug) | 0.0114 ± 0 .0025 | 0.0061 ± 0.0038 | | 0.0053 ± 0.001 | 2.135 | 0.127 |
| B3GALTL (pg/ug) | 5.88 ±1.32 | 5.98 ± 3.57 | | 3.789 ± .52 | 0.850 | 0.432 |
| HTRA1 (pg/ug) | 4.42 ± 1.26 | 5.25 ± 3 | | 2.65± 0.28 | 0.831 | 0.440 |
| LIPC (pg/ug) | 3.79 ± 1.38 | 6.25 ± 3.69 | | 1.94 ± 0.23 | 1.179 | 0.314 |
| TIMP3 (pg/ug) | 0.056 ± 0.01 | 0.092 ± 0.04 | | 0.036 ± 0.004 | 2.492 | 0.091 |
| SLC16A8 (pg/ug) | 0.765 ± 0.14 | 0.432 ± 0.32 | | 0.978 ± 0.14 | 1.165 | 0.319 |
|  | **APOE (rs 769449) Genotype** | | | |  |  |
|  | GG | | AG | | F-value | P-value |
| IER3 (pg/ug) | 3.55 ± 1.08 | | 2.4 ± 0.91 | | 0.190 | 0.665 |
| ADAMTS9 (pg/ug) | 4.61 ± 1.1 | | 4.89 ± 1.49 | | 0.011 | 0.918 |
| APOE (pg/ug) | 0.008 ± 0.001 | | 0.01 ± 0.004 | | 0.394 | 0.532 |
| B3GALTL (pg/ug) | 4.98 ± 0.87 | | 5.31 ± 0.84 | | 0.024 | 0.876 |
| HTRA1 (pg/ug) | 3.8 ± 0.80 | | 3.5 ± 0.53 | | 0.023 | 0.880 |
| LIPC (pg/ug) | 3.34 ± 0.89 | | 2.66 ± 0.65 | | 0.097 | 0.756 |
| TIMP3 (pg/ug) | 0.051 ± 0.007 | | 0.04 ± 0.008 | | 0.133 | 0.717 |
| SLC16A8 (pg/ug) | 0.76 ± 0.006 | | 0.69 ± 0.102 | | 0.069 | 0.794 |
|  | **B3GALTL (rs9542236) Genotype** | | | |  |  |
|  | TT | CC | | CT | F-value | P-value |
| IER3 (pg/ug) | 4.13 ± 1.53 | 3.12 ± 1.6 | | 2.15 ± 0.52 | 0.480 | 0.621 |
| ADAMTS9 (pg/ug) | 4.19 ± 0.52 | 1.52 ± 1.51 | | 4.95 ± 2.51 | 0.206 | 0.814 |
| APOE (pg/ug) | 0.009 ± 0.001 | 0.002 ± 0.0008 | | 0.004 ± 0.0007 | 1.938 | 0.153 |
| B3GALTL (pg/ug) | 5.91 ± 1.22 | 1.8 ± 0.60 | | 4.01 ± 0.49 | 0.978 | 0.382 |
| HTRA1 (pg/ug) | 4.4 ± 1.15 | 1.7 ± 0.30 | | 2.81 ± 0.31 | 0.686 | 0.507 |
| LIPC (pg/ug) | 3.87 ± 1.27 | 0.72 ± 0.36 | | 2.35 ± 0.38 | 0.571 | 0.568 |
| TIMP3 (pg/ug) | 0.053 ± 0.009 | 0.016 ± 0.007 | | 0.045 ± 0.008 | 0.534 | 0.589 |
| SLC16A8 (pg/ug) | 0.85 ± 0.12 | 1.45 ± 0.21 | | 0.72 ± 0.16 | 0.776 | 0.465 |
|  | **HTRA1 (rs11200638) Genotype** | | | |  |  |
|  | GG | AA | | GA | F-value | P-value |
| IER3 (pg/ug) | 3.5 ± 0.87 | 3.48 ± 1.74 | | 2.13 ± 0.56 | 0.175 | 0.840 |
| ADAMTS9 (pg/ug) | 3.71 ± 0.79 | 3.11 ± 0.35 | | 4.14 ± 1.11 | 0.686 | 0.508 |
| APOE (pg/ug) | 0.006 ± 0.001 | 0.006 ± 0.001 | | 0.008 ± 0.003 | 0.394 | 0.676 |
| B3GALTL (pg/ug) | 4.44 ± 0.95 | 5.11 ± 1.32 | | 4.19 ± 0.61 | 0.140 | 0.869 |
| HTRA1 (pg/ug) | 3.19 ± 0.51 | 3.81 ± 1.26 | | 3.01 ± 0.38 | 0.132 | 0.876 |
| LIPC (pg/ug) | 2.19 ± 0.43 | 3.49 ± 1.38 | | 2.02 ± 0.39 | 0.401 | 0.671 |
| TIMP3 (pg/ug) | 0.053 ± 0.01 | 0.047 ± 0.01 | | 0.04 ± 0.006 | 0.224 | 0.800 |
| SLC16A8 (pg/ug) | 0.54 ± 0.17 | 1.13 ± 0.14 | | 0.53 ± 0.15 | 4.818 | 0.011 |
|  | **TIMP3 (rs 5749482) Genotype** | | | |  |  |
|  | GG | | GC | | F-value | P-value |
| IER3 (pg/ug) | 3.4 ± 1.11 | | 3.17 ± 0.97 | | 0.008 | 0.928 |
| ADAMTS9 (pg/ug) | 5.12 ± 1.15 | | 2.7 ± 0.77 | | 0.911 | 0.343 |
| APOE (pg/ug) | 0.008 ± 0.001 | | 0.006 ± 0.002 | | 0.342 | 0.560 |
| B3GALTL (pg/ug) | 5.35 ± 0.85 | | 4.59 ± 1.5 | | 0.148 | 0.702 |
| HTRA1 (pg/ug) | 3.87 ± 0.79 | | 3.69 ± 1.27 | | 0.009 | 0.924 |
| LIPC (pg/ug) | 3.18 ± 0.87 | | 3.73 ± 1.56 | | 0.073 | 0.788 |
| TIMP3 (pg/ug) | 0.05 ± 0.007 | | 0.04 ± 0.014 | | 0.122 | 0.728 |
| SLC16A8 (pg/ug) | 0.82 ± 0.11 | | 0.69 ± 0.18 | | 0.229 | 0.634 |
|  | **IER3 (rs 3130783) Genotype** | | | |  |  |
|  | AA | GG | | AG | F-value | P-value |
| IER3 (pg/ug) | 3.12 ± 1.2 | 3.86 | | 4.75 ± 1.4 | 0.154 | 0.858 |
| ADAMTS9 (pg/ug) | 4.56 ± 1.2 | 7.77 | | 4.28 ± 0.98 | 0.082 | 0.921 |
| APOE (pg/ug) | 0.006 ± 0.001 | 0.019 | | 0.01 ± 0.003 | 1.406 | 0.253 |
| B3GALTL (pg/ug) | 4.65 ± 0.92 | 5.18 | | 6.83 ± 1.9 | 0.433 | 0.651 |
| HTRA1 (pg/ug) | 3.53 ± 0.87 | 3.86 | | 4.89 ± 1.6 | 0.194 | 0.824 |
| LIPC (pg/ug) | 3.23 ± 0.95 | 2.92 | | 3.33 ± 2.18 | 0.002 | 0.998 |
| TIMP3 (pg/ug) | 0.047 ± 0.007 | 0.05 | | 0.06 ± .01 | 0.157 | 0.855 |
| SLC16A8 (pg/ug) | 0.9 ± 0.11 | 0.96 | | 0.55 ± 0.21 | 0.813 | 0.448 |
|  | **SLC16A8 (rs 8135665) Genotype** | | | |  |  |
|  | CC | TT | | CT | F-value | P-value |
| IER3 (pg/ug) | 2.77 ± 0.59 | 0.88 ± 0.43 | | 4.52 ± 2.15 | 0.533 | 0.590 |
| ADAMTS9 (pg/ug) | 3.32 ± 0.37 | 1.11 ± 1.1 | | 4.55 ± 0.86 | 1.865 | 0.164 |
| APOE (pg/ug) | 0.006 ± 0.0009 | 0.002 ± 0.001 | | 0.01 ± 0.002 | 1.808 | 0.173 |
| B3GALTL (pg/ug) | 4.97 ± 0.65 | 2.86 ± 1.8 | | 5.81 ± 1.6 | 0.335 | 0.716 |
| HTRA1 (pg/ug) | 3.38 ± 0.51 | 2.38 ± 1.28 | | 4.54 ± 1.57 | 0.366 | 0.695 |
| LIPC (pg/ug) | 2.55 ± 0.65 | 2.47 ± 0.74 | | 4.39 ± 1.7 | 0.602 | 0.551 |
| TIMP3 (pg/ug) | 0.04 ± 0.005 | 0.03 ± 0.012 | | 0.06 ± 0.013 | 1.437 | 0.246 |
| SLC16A8 (pg/ug) | 1.03 ± 0.15 | 0.55 ± 0.55 | | 0.61 ± 0.13 | 2.100 | 0.132 |

**Table S2:** Expression of proteins on respective genotype in Indian AMD.
